# Aging-related volume changes in the brain and cerebrospinal fluid using artificial intelligence-automated segmentation

**DOI:** 10.1007/s00330-023-09632-x

**Published:** 2023-04-15

**Authors:** Shigeki Yamada, Tomohiro Otani, Satoshi Ii, Hiroto Kawano, Kazuhiko Nozaki, Shigeo Wada, Marie Oshima, Yoshiyuki Watanabe

**Affiliations:** 1 Department of Neurosurgery, Nagoya City University Graduate School of Medical Science, 1 Kawasumi, Mizuho-cho, Mizuho-ku, NagoyaNagoya, Aichi 467-8601 Japan; 2 Interfaculty Initiative in Information Studies / Institute of Industrial Science, The University of Tokyo, Tokyo, Japan; 3 Department of Neurosurgery, Shiga University of Medical Science, Ōtsu, Shiga Japan; 4 Department of Mechanical Science and Bioengineering, Graduate School of Engineering Science, Osaka University, Osaka, Japan; 5 Faculty of System Design, Tokyo Metropolitan University, Hachioji, Tokyo Japan; 6 Department of Radiology, Shiga University of Medical Science, Ōtsu, Shiga Japan

**Keywords:** Aging, Artificial intelligence, Cerebrospinal fluid, Cerebral ventricle, Subarachnoid space

## Abstract

**Objectives:**

To verify the reliability of the volumes automatically segmented using a new artificial intelligence (AI)-based application and evaluate changes in the brain and CSF volume with healthy aging.

**Methods:**

The intracranial spaces were automatically segmented in the 21 brain subregions and 5 CSF subregions using the AI-based application on the 3D T1-weighted images in healthy volunteers aged > 20 years. Additionally, the automatically segmented volumes of the total ventricles and subarachnoid spaces were compared with the manually segmented volumes of those extracted from 3D T2-weighted images using the intra-class correlation and Bland–Altman analysis.

**Results:**

In this study, 133 healthy volunteers aged 21–92 years were included. The mean intra-class correlations between the automatically and manually segmented volumes of the total ventricles and subarachnoid spaces were 0.986 and 0.882, respectively. The increase in the CSF volume was estimated to be approximately 30 mL (2%) per decade from 265 mL (18.7%) in the 20s to 488 mL (33.7%) in ages above 80 years; however, the increase in the volume of total ventricles was approximately 20 mL (< 2%) until the 60s and increased in ages above 60 years.

**Conclusions:**

This study confirmed the reliability of the CSF volumes using the AI-based auto-segmentation application. The intracranial CSF volume increased linearly because of the brain volume reduction with aging; however, the ventricular volume did not change until the age of 60 years and above and then gradually increased. This finding could help elucidate the pathogenesis of chronic hydrocephalus in adults.

**Key Points:**

• *The brain and CSF spaces were automatically segmented using an artificial intelligence-based application*.

• *The total subarachnoid spaces increased linearly with aging, whereas the total ventricle volume was around 20 mL (< 2%) until the 60s and increased in ages above 60 years.*

• *The cortical gray matter gradually decreases with aging, whereas the subcortical gray matter maintains its volume, and the cerebral white matter increases slightly until the 40s and begins to decrease from the 50s.*

## Introduction

Brain atrophy defined as a loss of neurons and connecting fibers is caused by aging and several neurodegenerative disorders. As a representative disease, Alzheimer’s disease is characterized by atrophy of the medial temporal lobe including the hippocampus [1–4]. Several software for automatic computer-aided brain segmentation have been used as a medical device that assists in diagnosing Alzheimer’s disease. For example, the voxel-based specific regional analysis system for Alzheimer’s disease (VSRAD) software has been widely used as a reference for diagnostic imaging of Alzheimer’s disease in Japan [5, 6]. Additionally, age-related volume changes in the gray and white matters and cerebrospinal fluid (CSF) segmented using manual or voxel-based morphometry methods have been investigated [7–16]. Our previous volumetric study on three-dimensional (3D) T2-weighted images reported that the mean volume of intracranial CSF in healthy volunteers aged 60 years or older was more than 330 mL, of which the ventricular volume was more than 60 mL and the subarachnoid space volume was approximately 270 mL [17–19]. It is natural that the total volume of intracranial CSF increases as a compensation for brain volume decreases with aging. However, many studies and reviews have reported that the mean volume of intracranial CSF is approximately 150 mL with 25 mL in the ventricles and 125 mL in the subarachnoid spaces in adults [20–22]. Recently, the automatic method for segmenting the brain from 3D MRI has rapidly shifted from voxel-based morphometry to deep learning based on convolutional neural networks. For example, deep learning methods for measuring the medial temporal lobe or hippocampus have been reported to be useful for predicting or diagnosing Alzheimer’s disease [23–26]. A new application named Brain Subregion Analysis launched in the SYNAPSE 3D workstation (FUJIFILM Corporation), which has been the most widely used workstation (approximately 1300 facilities) in Japan, was released in 2020. In this application, the brain and CSF in the skull on 3D T1-weighted images are automatically segmented into 21 brain subregions and 5 CSF subregions within 1 min using deep learning. This study was designed to verify the reliability of the automatically segmented CSF volumes and investigate trends in volume reduction of the segmented brain and compensatory increases in CSF volume because of healthy aging.

## Materials and methods

### Ethical approvals

The design and protocol of this study were approved by the Ethics Committee for Human Research of our institution (IRB number: R2019-227). Healthy volunteers underwent MRI after providing written informed consent, and we particularly explained the potential for detecting diseases in the brain. MRI data were extracted after the private information of the volunteers was anonymized in a linkable manner. The study design was prospective and observational. This study was conducted according to the approved guidelines of the Declaration of Helsinki.

### Study population

From November 2020 to April 2022, approximately 20 or more healthy volunteers aged ≥ 20 years with no upper age limit for every decade were recruited from medical staff and their families by open recruitment. The inclusion criteria for this study were as follows: participants who had no history of brain injury, brain tumor, or cerebrovascular disease on previous brain MRI or those who had never undergone brain CT or MRI and no neurological symptoms. The exclusion criteria were as follows: the artifacts in the head, particularly dentures, were large and affected the MRI results. After brain MRI, three volunteers were incidentally determined to have small unruptured intracranial aneurysms with a maximum diameter of < 2 mm. They were included in this study because small unruptured aneurysms might not affect the volumes of the brain and CSF.

### Image acquisitions

All MRI examinations were performed using a 3-tesla MRI machine (Signa Architect 3.0T or Discovery MR 750W, GE Healthcare). The 3D T1-weighted magnetization-prepared rapid gradient-echo (MPRAGE) and 3D T2-weighted fast spin-echo Cube sequences were obtained in a sagittal orientation. The sequence parameters for MPRAGE were as follows: repetition time (TR), 2,471 ms; time to echo (TE), 3.13 ms; inversion time, 1,000 ms; flip angle, 8°; matrix 256 × 256; voxel size, 0.9 × 0.9 × 0.9 mm; and acquisition time, approximately 4 min. The sequence parameters for Cube were as follows: TR, 2,000 ms; TE, 85.3 ms; matrix 288 × 288; voxel size, 0.8 × 0.8 × 0.8 mm; and acquisition time, approximately 4 min.

### Data processing

**Fig. 1:**
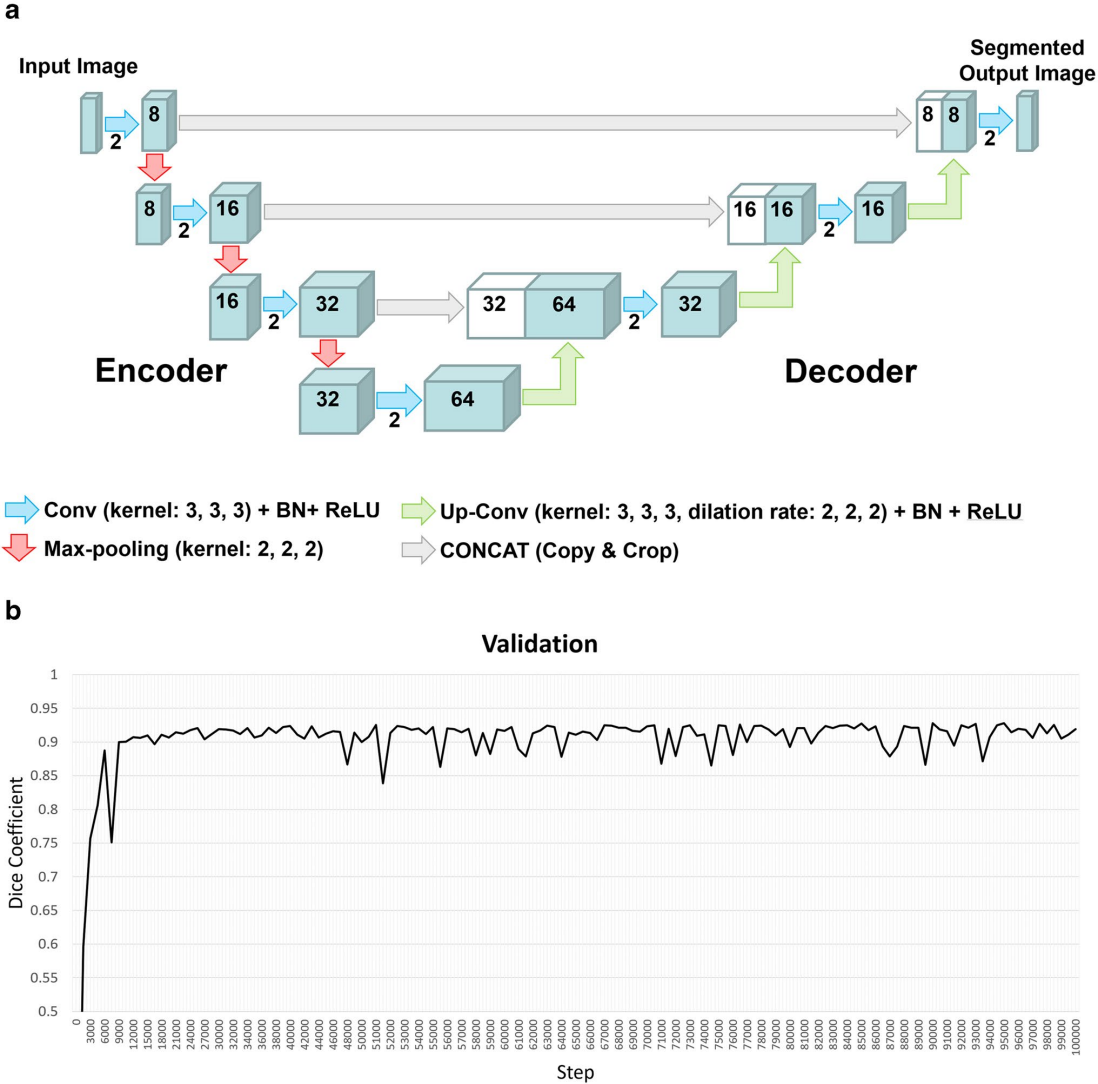
3D U-Net model with four layers and validation result for Brain Subregion Analysis application. **a** Each blue box corresponds to a multi-channel feature map. The number of channels is denoted on top of the box. White boxes indicate copied feature maps. The color arrows indicate each process: sky blue arrows indicate convolution (Conv) with kernel size (3, 3, 3) in addition to batch normalization (BN) and rectified linear unit (ReLU) activation layer, red arrows indicate max-pooling with kernel size (2, 2, 2), green arrows indicate up-convolution (Up-Conv) with kernel size (3, 3, 3) and dilation rate (2, 2, 2) in addition to BN and ReLU, and gray arrows indicate direct concatenation from each encoding layer of feature map extracted by downsampling to the corresponding decoding layer of feature map by upsampling. **b** Dice coefficients for each validation step of the 3D U-Net model. The dice coefficient exceeded 0.9 at 10,000 steps and remained consistently high until 100,000 steps

The Brain Subregion Analysis application on an independent 3D volume analyzer workstation (SYNAPSE 3D; FUJIFILM Corporation) was approved as a medical device by the Pharmaceuticals and Medical Devices Agency of Japan in 2020. It uses a novel image recognition technology based on Fujifilm’s AI-enabled platform REiLI to accurately recognize and consistently extract specific regions of the brain. As training data for deep learning, the Alzheimer’s Disease Neuroimaging Initiative (ADNI) database (adni.loni.usc.edu) was used [23–26]. The ADNI MRI dataset included 653 individuals, including 255 healthy individuals, 179 individuals diagnosed with mild cognitive impairment, and 219 individuals diagnosed with Alzheimer’s disease. In addition, 5 healthy individuals from ADNI data and 29 individuals from in-house data were used for validation. The region segmentation maps for the training and validation data sets were created manually, referring to the publicly available Mindboggle atlas (https://mindboggle.info/) and checking with a neuroradiologist. The deep learning model employed a 3D U-Net structure, consisting of 3D convolution, batch normalization, ReLU activation layer, max pooling layer, and 3D up-convolution (Fig. 1A). At the end of the last convolutional layer, the final features were fed to a softmax activation function to generate probability scores for each class. The dice coefficient was used for loss function in the deep learning. The image intensities of the input images were normalized to [0, 1] by their maximum and minimize values. Batch size for training was 4, and optimaiza was set at a learning rate of 0.0001 in Adam. To improve generalizability and segmentation accuracy, the following augmentation was randomly performed for each input image: similar transformation with rotation (0–15 degrees in each axis), scaling (0.95–1.05 times) and translation (0–5%), flipping, sharpness or blur filter with standard deviations (SDs) of 0.5–2, Gaussian noise with SDs of 0–0.05, image intensity shifting (± 0–10% of the signal range), and perturbation (± 0–15% of the signal range). These augmentations can reduce the effects of differences in manufacturers, imaging protocol, or individuals. As a result of deep learning up to 100,000 steps, the feature map at the step with the best validation result was finally adopted for the Brain Subregion Analysis application, with the highest average dice coefficient of 0.928, accuracy of 0.998, precision of 0.921, and repeatability of 0.934 for the 26 regions (Fig. 1B).

### Segmentation and measurement

**Fig. 2:**
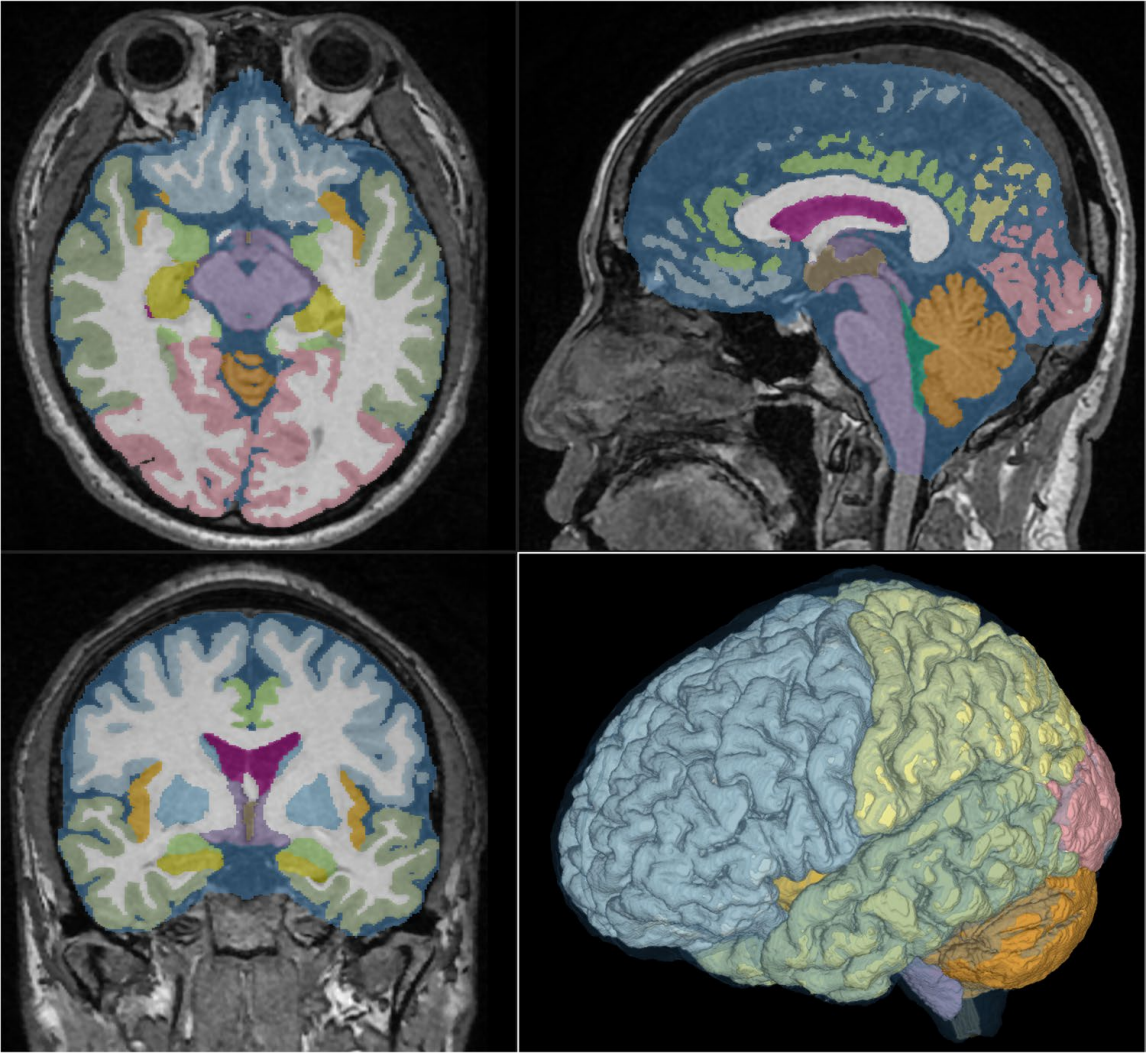
Screenshot of automatic segmentation using Brain Subregion Analysis application. This screenshot shows the result of a 48-year-old male healthy brain on the Brain Subregion Analysis application of the 3D volume analyzer SYNAPSE 3D workstation (FUJIFILM Corporation). By default, the axial view is displayed in the upper left, the sagittal view is in the upper right, the coronal view is in the lower left, and 3D is in the lower right

Using the Brain Subregion Analysis application, the brain on the 3D T1-weighted MPRAGE sequence was automatically segmented, and their segmented volumes were quantified within approximately 1 min in the following 21 brain subregions: the frontal cortex; parietal cortex; temporal cortex; occipital cortex; insular cortex; cerebral white matter; hippocampus, including the parahippocampal gyrus (entorhinal cortex); basal ganglia, including the caudate nucleus; putamen; globus pallidus; limbic system, including the cingulate gyrus and amygdala; brainstem, including the thalamus, hypothalamus, midbrain, pons, and medulla oblongata; and cerebellum (Fig. 2, Videoclip in Supplementary Material). In this study, cortical gray matter was defined as the combined region of the frontal, temporal, parietal, occipital, and insular cortex, and subcortical gray matter was defined as the combined region of the hippocampus, basal ganglia, and brainstem. Additionally, CSF spaces were divided into the following five subregions: the bilateral lateral ventricles, third ventricle, fourth ventricle, and subarachnoid spaces.

To evaluate the reliability of the volumes segmented using the Brain Subregion Analysis application on the 3D T1-weighted MPRAGE sequence, the total ventricles and subarachnoid spaces in the same volunteers were segmented from the 3D T2-weighted Cube sequence in our original method, combining a simple threshold algorithm and manual segmentation, as previously reported [17–19]. The intra reliability of the AI segmentation method is perfect because it is fully automated. In contrast, our original segmentation method takes time to segment, and the reproducibility is not high. The reliability and validity of the segmented CSF volumes measured using our original method were assessed and described in a previous article [17–19].

### Statistical analysis

The sex and left–right differences in the segmented volumes and volume ratios, which were defined as the volume divided by intracranial volume, were compared using the Mann–Whitney–Wilcoxon test. The volunteers were divided into the following three subgroups according to their ages at the time of MRI examination: under 40 years old, 40–59 years old, and 60 years old and above. The mean volumes and volume ratios ± SDs in three age subgroups were compared using the Kruskal–Wallis rank sum test. The chi-square test was used to compare the proportions of the groups. To evaluate the consistency of the volumes measured using the AI-based and manual segmentation methods, the intra-class correlation (ICC) for two-way models and Bland–Altman analysis were applied. An ICC of ≥ 0.9 was interpreted as excellent reliability, 0.8–0.9 as good, 0.7–0.8 as acceptable, 0.6–0.7 as questionable, and < 0.6 as poor. The difference (bias) between the segmented volumes was defined as significant when the distribution of the difference was not within the 1.96 ± SD of the mean difference in the Bland–Altman plot. A small bias within the acceptable range was defined as an average difference in volume measured using two different methods that was less than 10% of the original volume. The relationships between segmented volumes or volume ratios and age were examined using Pearson’s correlation coefficient (*r*) and 95% confidential intervals (CIs). Additionally, statistical significance was assumed at a probability (*P*) value of < 0.05. All missing data points were treated as deficit data that did not affect other variables. Statistical analyses were performed using R (version 4.1.1; The R Foundation for Statistical Computing; http://www.R-project.org).

## Results

### Clinical characteristics and sex difference

In this study, 133 healthy volunteers (mean age, 43.9 ± 14.7 years; range, 21–92 years; 46 males and 87 females) were included. We recruited healthy senior volunteers in their 70s and older, but were unable to attract a sufficient number because many seniors had some imaging findings or physical symptoms. Therefore, those aged 60 or older were categorized into one age group for comparison. Males had significantly larger intracranial volumes and segmented volumes of the brain and CSF than females. However, all volume ratios divided by their intracranial volume were not significantly different between males and females, except for the parietal cortex, cerebellum, lateral ventricle, and third ventricle (Table 1).

**Table 1:** Differences in the volume and volume ratio of the segmented regions according to sex

	All	Female	Male	*p* value
Total number	133	87	46	
Age (years)	47.7 ± 16.8	46.7 ± 16.2	49.8 ± 17.9	0.372
20s	22	16	6	
30s	25	15	10	
40s	24	14	10	
50s	25	20	5	
60s	22	15	7	
70s	13	7	6	
80s <	2	0	2	
Intracranial space (volume, mL)	1443.3 ± 129.0	1393.2 ± 91.2	1538.2 ± 137.3	< 0.001
Total brain (mL)	1100.3 ± 113.3	1067.7 ± 88.1	1162.1 ± 129.9	< 0.001
Cortical gray matter (mL)	457.6 ± 50.0	445.6 ± 43.9	480.4 ± 53.4	< 0.001
Frontal cortex (mL)	168.0 ± 19.2	163.8 ± 17.3	176.0 ± 20.3	0.002
Parietal cortex (mL)	115.0 ± 12.7	112.5 ± 11.9	119.8 ± 13.1	0.006
Temporal cortex (mL)	118.5 ± 14.1	115.0 ± 11.9	125.1 ± 15.7	< 0.001
Occipital cortex (mL)	56.1 ± 7.3	54.3 ± 6.4	59.5 ± 7.8	< 0.001
Cerebral white matter (mL)	417.9 ± 50.9	405.0 ± 38.0	442.3 ± 62.4	< 0.001
Cerebellum (mL)	135.8 ± 13.8	132.6 ± 11.7	141.8 ± 15.5	< 0.001
Brainstem (mL)	42.7 ± 3.8	41.4 ± 3.2	45.0 ± 3.7	< 0.001
Insula cortex (mL)	12.8 ± 1.4	12.3 ± 1.2	13.7 ± 1.4	< 0.001
Hippocampus (mL)	6.8 ± 0.7	6.6 ± 0.5	7.2 ± 0.8	< 0.001
Basal ganglia (mL)	13.6 ± 1.5	13.2 ± 1.3	14.4 ± 1.5	< 0.001
Limbic system (mL)	33.2 ± 3.8	32.2 ± 3.3	35.3 ± 4.0	< 0.001
Total ventricle (mL)	25.2 ± 12.2	22.6 ± 10.4	30.1 ± 13.8	< 0.001
Lateral ventricle (mL)	22.6 ± 11.6	20.2 ± 10.0	27.1 ± 13.0	< 0.001
Third ventricle (mL)	1.0 ± 0.6	0.9 ± 0.5	1.3 ± 0.7	< 0.001
Fourth ventricle (mL)	1.6 ± 0.4	1.5 ± 0.3	1.7 ± 0.4	0.030
Total subarachnoid space (mL)	297.7 ± 55.9	281.6 ± 42.3	328.1 ± 65.7	< 0.001
Intracranial CSF space (mL)	322.9 ± 64.9	304.2 ± 49.2	358.2 ± 76.1	< 0.001
Total ventricle on 3D T2 (mL)	26.9 ± 12.1	24.5 ± 10.6	31.6 ± 13.5	< 0.001
Total subarachnoid space on 3D T2 (mL)	251.1 ± 62.0	232.6 ± 51.2	286.7 ± 66.1	< 0.001
Intracranial CSF space on 3D T2 (mL)	278.0 ± 70.0	257.2 ± 57.5	318.3 ± 74.8	< 0.001
Total brain (volume ratio, %)	76.2 ± 3.4	76.6 ± 3.0	75.5 ± 4.1	0.176
Cortical gray matter (%)	31.7 ± 2.0	32.0 ± 1.9	31.2 ± 2.0	0.058
Frontal cortex (%)	11.6 ± 0.8	11.7 ± 0.8	11.4 ± 0.8	0.073
Parietal cortex (%)	8.0 ± 0.6	8.1 ± 0.6	7.8 ± 0.5	0.013
Temporal cortex (%)	8.2 ± 0.6	8.2 ± 0.6	8.1 ± 0.7	0.350
Occipital cortex (%)	3.9 ± 0.3	3.9 ± 0.4	3.9 ± 0.3	0.665
Cerebral white matter (%)	28.9 ± 2.0	29.1 ± 1.6	28.7 ± 2.5	0.485
Cerebellum (%)	9.4 ± 0.8	9.5 ± 0.7	9.2 ± 0.8	0.029
Brainstem (%)	3.0 ± 0.2	3.0 ± 0.2	2.9 ± 0.2	0.123
Insula cortex (%)	0.89 ± 0.06	0.88 ± 0.06	0.89 ± 0.06	0.769
Hippocampus (%)	0.48 ± 0.03	0.48 ± 0.03	0.47 ± 0.04	0.271
Basal ganglia (%)	0.94 ± 0.08	0.95 ± 0.07	0.94 ± 0.09	0.936
Limbic system (%)	2.3 ± 0.1	2.3 ± 0.1	2.3 ± 0.1	0.645
Total ventricle (%)	1.7 ± 0.8	1.6 ± 0.7	1.9 ± 0.8	0.017
Lateral ventricle (%)	1.5 ± 0.7	1.4 ± 0.7	1.7 ± 0.8	0.018
Third ventricle (%)	0.07 ± 0.04	0.06 ± 0.03	0.08 ± 0.04	0.008
Fourth ventricle (%)	0.11 ± 0.02	0.11 ± 0.02	0.11 ± 0.03	0.867
Intracranial CSF space (%)	22.4 ± 3.9	21.9 ± 3.5	23.3 ± 4.6	0.131

*p-*values of the sex difference based on the Mann–Whitney–Wilcoxon test

### Reliability of segmentation

**Fig. 3:**
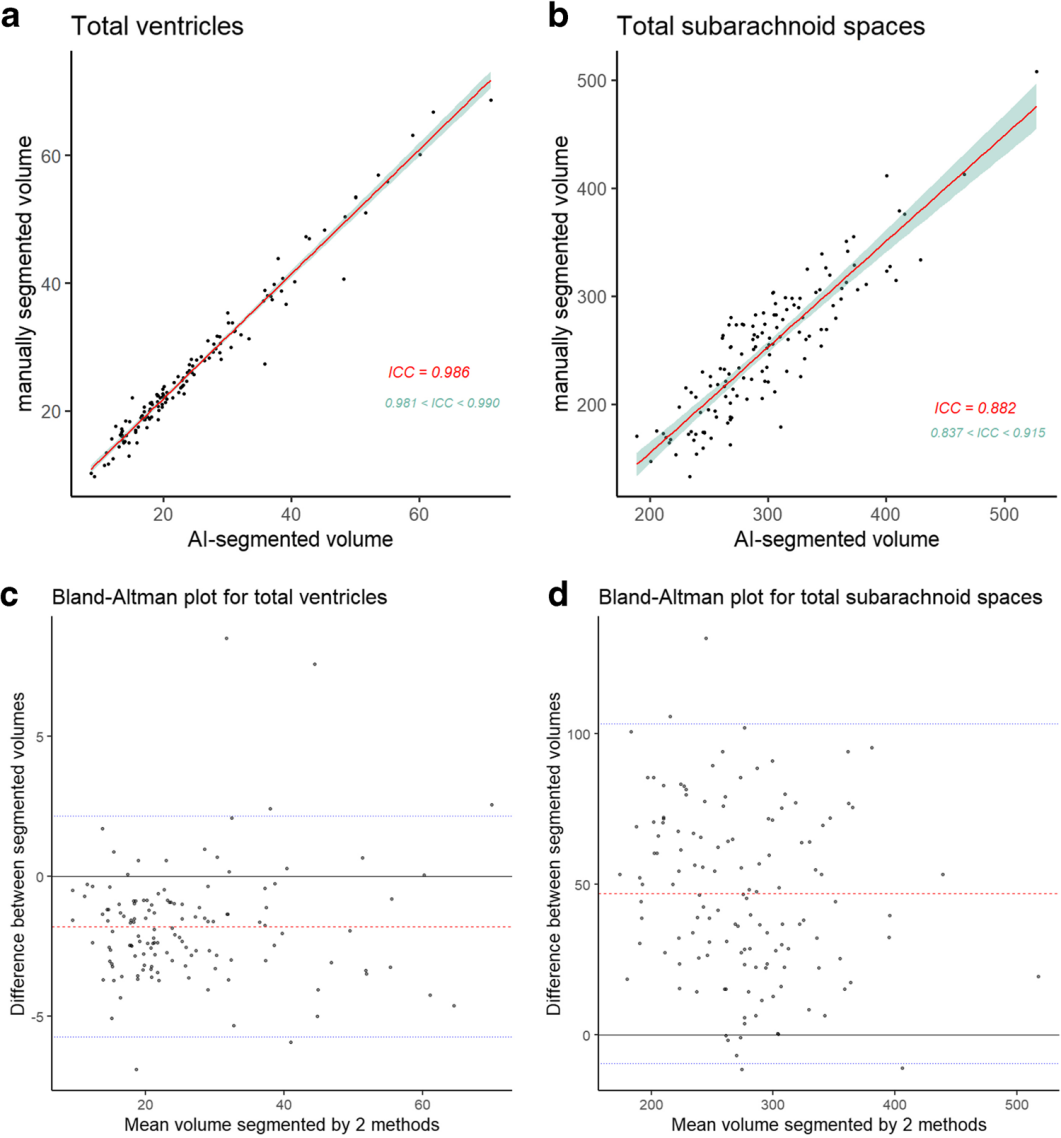
Reliability of the cerebrospinal fluid volumes segmented using two methods. The upper graphs show the scatterplots and linear regression lines (red) with 95% confidential intervals (green) of the segmented volumes of the total ventricles (**a**) and subarachnoid spaces (**b**) using the two methods. The lower graphs show the Bland–Altman analysis for the segmented volumes of the total ventricles (**c**) and subarachnoid spaces (**d**) using the two methods

**Fig. 4:**
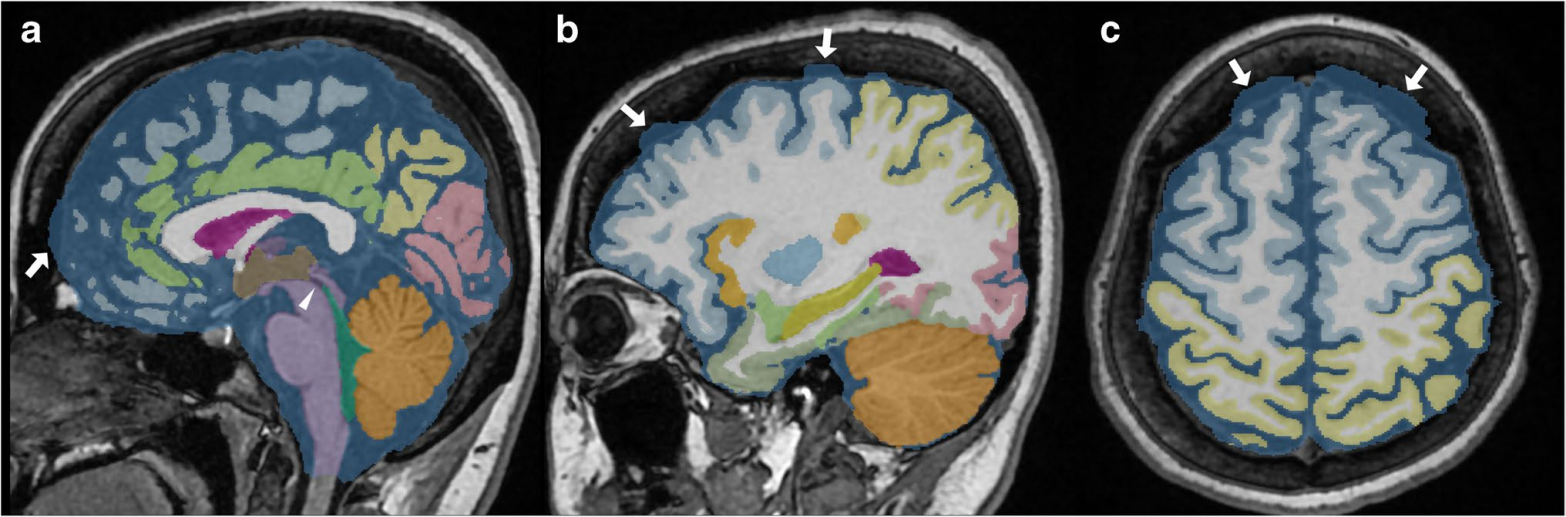
A case of failed segmentation of ventricles and subarachnoid spaces. The cerebral aqueduct, the boundary between the third and fourth ventricles, was missing (white arrowhead). The subarachnoid space at the convexity region contained some parts of the inner plate of the skull (white arrow). **a** Sagittal midplane, (**b**) sagittal section passing through the right eye center, (**c**) axial section in the high-convexity part

The total volume of the lateral, third, and fourth ventricles segmented using the AI-based automated segmentation method on the 3D T1-weighted MPRAGE sequence had an excellent agreement with the total ventricular volume manually segmented using our original method on the 3D T2-weighted Cube sequence (mean ICC, 0.986; 95% CI, 0.981–0.990) (Fig. 3A). The volume of the total subarachnoid space also showed a good reliability (mean ICC, 0.882; 95% CI, 0.837–0.915) (Fig. 3B). The total ventricular volume using the AI-based segmentation method was 1.7 mL smaller on average (25.2 mL vs. 26.9 mL) than that using the manual segmentation method (Fig. 3C), whereas the total subarachnoid space volume was 46.6 mL larger (297.7 mL vs. 251.1 mL, Fig. 3D). The systematic error in ventricle segmentation was caused by the loss of ventricular boundaries in the AI-based segmentation method, while that in subarachnoid space segmentation was caused by the inclusion of part of the inner plate of the skull in the superficial subarachnoid spaces (Fig. 4).

### Laterality and age-related volume changes

Although the total brain volumes on the right and left sides were almost the same, the volumes and volume ratios of the frontal cortex, insula cortex, and hippocampus were significantly smaller on the left side, and those of the basal ganglia, limbic system, and lateral ventricle were significantly larger on the left side than on the right side (Table 2).

**Table 2:** Left–right difference in the volume and volume ratio of the segmented regions

	Left	Right	Difference	*p* value
Total brain (mL)	551.1 ± 56.9	549.2 ± 56.7	1.9 ± 7.2	0.705
Frontal cortex (mL)	82.3 ± 9.4	85.7 ± 10.0	−3.5 ± 2.7	0.004
Parietal cortex (mL)	57.7 ± 6.5	57.3 ± 6.4	0.4 ± 1.9	0.674
Temporal cortex (mL)	59.4 ± 7.1	59.1 ± 7.2	0.2 ± 2.0	0.738
Occipital cortex (mL)	27.5 ± 3.6	28.6 ± 4.0	−1.0 ± 2.2	0.040
Cerebral white matter (mL)	208.8 ± 25.3	209 ± 25.6	−0.2 ± 3.0	0.943
Cerebellum (mL)	68.4 ± 7.2	67.4 ± 6.6	1.0 ± 1.6	0.201
Insula cortex (mL)	6.3 ± 0.7	6.5 ± 0.7	−0.3 ± 0.3	0.002
Hippocampus (mL)	3.3 ± 0.3	3.5 ± 0.4	−0.2 ± 0.1	< 0.001
Basal ganglia (mL)	7 ± 0.8	6.6 ± 0.8	0.4 ± 0.3	< 0.001
Limbic system (mL)	18.1 ± 2.1	15.1 ± 1.8	3.0 ± 1.2	< 0.001
Lateral ventricle (mL)	12.3 ± 6.2	10.3 ± 5.6	2.0 ± 2.3	< 0.001
Total brain (volume ratio, %)	38.2 ± 1.7	38.0 ± 1.7	0.1 ± 0.5	0.607
Frontal cortex (%)	5.7 ± 0.4	5.9 ± 0.4	−0.2 ± 0.2	< 0.001
Parietal cortex (%)	4.0 ± 0.3	4.0 ± 0.3	0.03 ± 0.13	0.444
Temporal cortex (%)	4.1 ± 0.3	4.1 ± 0.3	0.01 ± 0.14	0.744
Occipital cortex (%)	1.9 ± 0.2	2.0 ± 0.2	−0.07 ± 0.15	0.003
Cerebral white matter (%)	14.5 ± 1.0	14.5 ± 1.0	−0.01 ± 0.21	0.880
Cerebellum (%)	4.7 ± 0.4	4.7 ± 0.4	0.07 ± 0.11	0.119
Insula cortex (%)	0.43 ± 0.03	0.45 ± 0.03	−0.02 ± 0.02	< 0.001
Hippocampus (%)	0.23 ± 0.01	0.25 ± 0.02	−0.02 ± 0.01	< 0.001
Basal ganglia (%)	0.49 ± 0.04	0.46 ± 0.04	0.03 ± 0.02	< 0.001
Limbic system (%)	1.3 ± 0.1	1.1 ± 0.1	0.2 ± 0.1	< 0.001
Lateral ventricle (%)	0.8 ± 0.4	0.7 ± 0.4	0.1 ± 0.2	< 0.001

*p-*value of the right–left difference based on the Mann–Whitney–Wilcoxon test

**Fig. 5:**
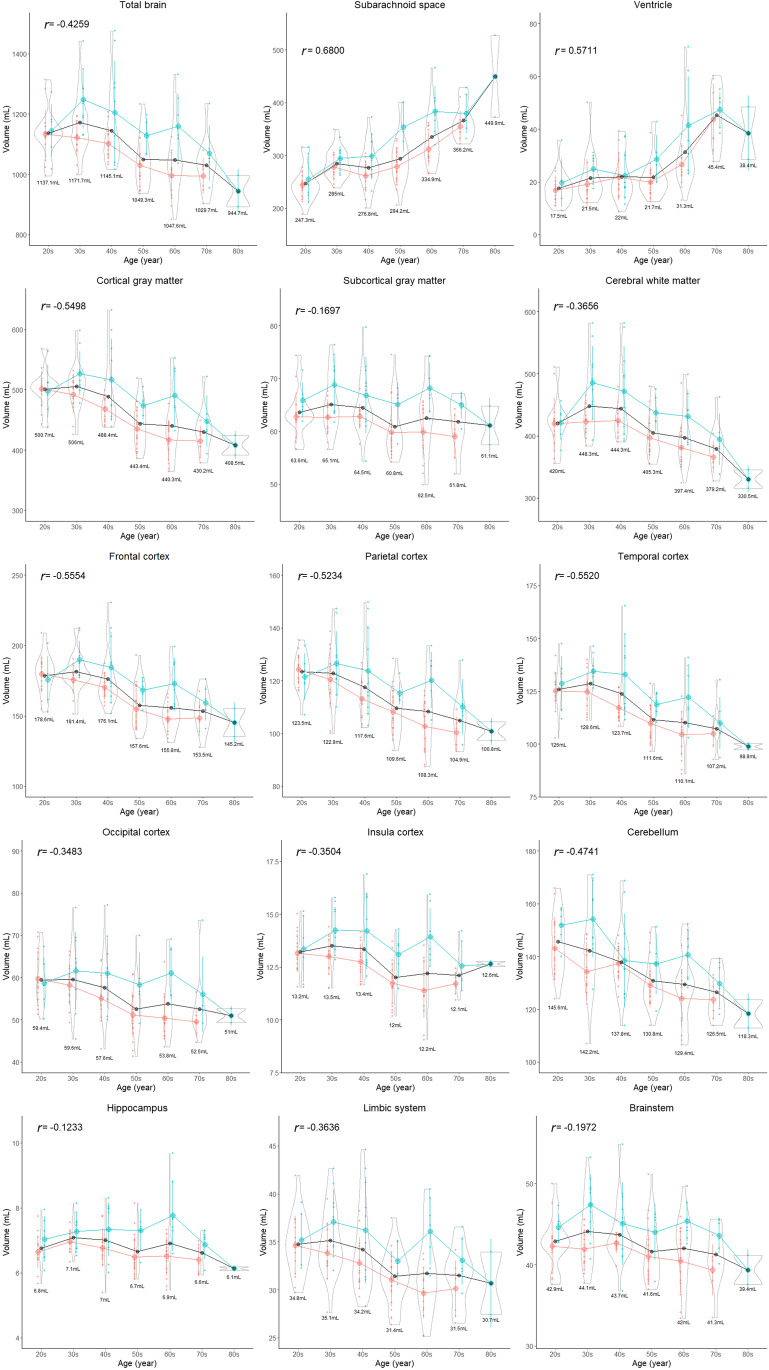
Segmented region volume. Each graph is a combination of violin plots for the distribution of the segmented volume and line graphs for the mean volume in each decade stratified by sex. Red indicates female, blue indicates male, and black indicates all. The red and blue vertical lines contain the volumes between the 25th and 75th percentiles. The mean volume in each decade is shown under the violin plot. The relationship between segmented volume and age in each region was examined using Pearson’s correlation coefficient (*r*)

**Fig. 6:**
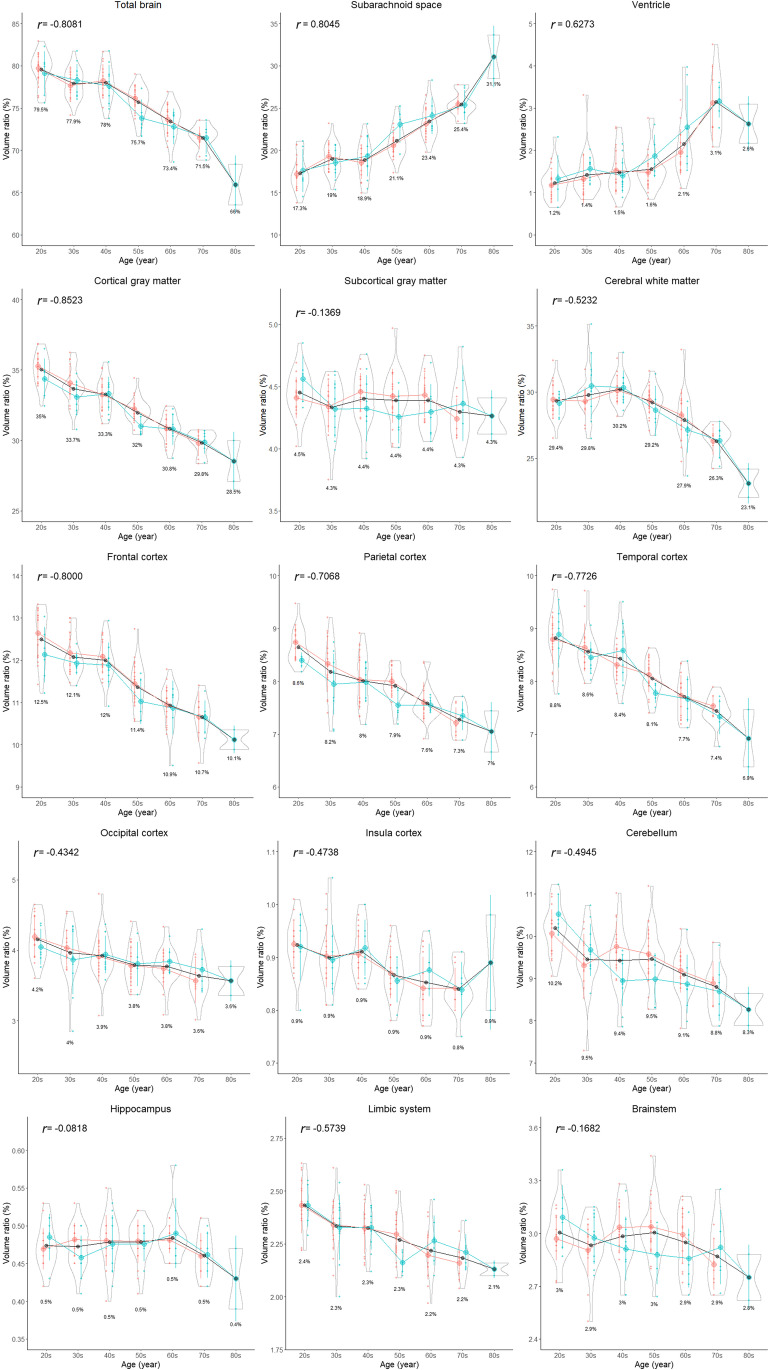
Segmented region volume ratio. Each graph is a combination of violin plots for the distribution of the segmented volume ratio and line graphs for the mean volume ratio in each decade stratified by sex. Red indicates female, blue indicates male, and black indicates all. The red and blue vertical lines contain the volume ratios between the 25th and 75th percentiles. The mean volume ratio in each decade is shown under the violin plot. The relationship between segmented volume ratio and age in each region was examined using Pearson’s correlation coefficient (*r*)

 All segmented volumes and volume ratios, except for the hippocampus, basal ganglia, brain stem, and fourth ventricle, were significantly different among the three age groups (Table 3). All parts of the cortex were significantly and gradually smaller, whereas the lateral and third ventricles and subarachnoid spaces were significantly larger in the older group than in the younger group. The distributions of the segmented volumes and volume ratios for each age stratified according to sex are shown in Figs. 5 and 6, respectively. With aging, the volume and volume ratio of the entire brain linearly decrease, and those of the total intracranial CSF linearly increase. The increase ratio in the CSF volume (volume ratio) was estimated to be approximately 30 mL (2%) per decade, from 265 mL (18.7%) in the 20s to 488 mL (33.7%) in ages above 80 years. However, the increase ratio in the ventricular volume with aging was not constant compared with that in the subarachnoid space. The volume and volume ratio of the total ventricle were approximately 20 mL and < 2% on average until the 60s and increase in ages above 60 years. The cortical gray matter gradually decreased with aging, whereas the subcortical gray matter maintained its volume, and the cerebral white matter increased slightly until the 40s and began to decrease from the 50s. The volume ratio of the cortical gray matter had the strongest negative correlation with age (*r*: −0.852; 95% CI: −0.893 to −0.789), and that of the total CSF had the strongest positive correlation (*r*: 0.824; 95% CI, 0.760–0.872), although those of the subcortical gray matter, hippocampus, basal ganglia, and brain stem were not significantly associated with age (Figs. 5 and 6).

**Table 3:** Differences in the volume and volume ratio of the segmented regions among the three age groups

	< 40 years	40–59 years	≥60 years	*p* value
Total number (female:male)	47 (31:16)	49 (34:15)	37 (22:15)	
Intracranial space (volume, mL)	1468.7 ± 105.7	1427.0 ± 141.0	1432.7 ± 137.5	0.066
Total brain (mL)	1155.5 ± 90.8	1096.2 ± 115.3	1035.8 ± 102.8	< 0.001
Cortical gray matter (mL)	490.2 ± 33.6	452.7 ± 48.4	422.8 ± 44.1	< 0.001
Frontal cortex (mL)	180.1 ± 13.5	166.7 ± 19.1	154.4 ± 15.8	< 0.001
Parietal cortex (mL)	123.1 ± 9.5	113.5 ± 11.9	106.7 ± 11.4	< 0.001
Temporal cortex (mL)	127.4 ± 9.6	117.5 ± 13.6	108.5 ± 12.6	< 0.001
Occipital cortex (mL)	59.5 ± 6.4	55.0 ± 7.2	53.2 ± 7.0	< 0.001
Cerebral white matter (mL)	435.1 ± 47.2	424.4 ± 50.2	387.4 ± 43.4	< 0.001
Cerebellum (mL)	143.8 ± 13.8	134.2 ± 11.3	127.8 ± 11.4	< 0.001
Brainstem (mL)	43.5 ± 3.6	42.6 ± 3.7	41.6 ± 3.8	0.166
Insula cortex (mL)	13.4 ± 1.1	12.7 ± 1.5	12.2 ± 1.5	< 0.001
Hippocampus (mL)	6.9 ± 0.5	6.8 ± 0.7	6.8 ± 0.8	0.302
Basal ganglia (mL)	13.9 ± 1.4	13.2 ± 1.4	13.8 ± 1.6	0.035
Limbic system (mL)	35.0 ± 3.2	32.8 ± 3.9	31.6 ± 3.7	< 0.001
Total ventricle (mL)	19.6 ± 7.5	21.9 ± 8.0	36.6 ± 14.1	< 0.001
Lateral ventricle (mL)	17.3 ± 7.2	19.4 ± 7.5	33.4 ± 13.4	< 0.001
Third ventricle (mL)	0.8 ± 0.3	0.9 ± 0.4	1.5 ± 0.7	< 0.001
Fourth ventricle (mL)	1.6 ± 0.4	1.5 ± 0.4	1.7 ± 0.4	0.078
Total subarachnoid space (mL)	267.3 ± 35.0	285.7 ± 43.5	352.1 ± 54.3	< 0.001
Intracranial CSF space (mL)	287.0 ± 38.3	307.6 ± 47.8	388.7 ± 63.8	< 0.001
Total ventricle on 3D T2 (mL)	21.5 ± 7.1	23.7 ± 8.2	38.0 ± 14.1	< 0.001
Total subarachnoid space on 3D T2 (mL)	215.2 ± 43.5	243.5 ± 51.9	305.6 ± 57.2	< 0.001
Intracranial CSF space on 3D T2 (mL)	236.7 ± 47.0	267.2 ± 55.6	343.6 ± 64.5	< 0.001
Total brain (volume ratio, %)	78.7 ± 2.0	76.8 ± 2.3	72.3 ± 2.6	< 0.001
Cortical gray matter (%)	33.4 ± 1.4	31.7 ± 1.2	29.5 ± 1.1	< 0.001
Frontal cortex (%)	12.3 ± 0.6	11.7 ± 0.6	10.8 ± 0.5	< 0.001
Parietal cortex (%)	8.4 ± 0.5	8.0 ± 0.4	7.4 ± 0.4	< 0.001
Temporal cortex (%)	8.7 ± 0.5	8.2 ± 0.4	7.6 ± 0.4	< 0.001
Occipital cortex (%)	4.1 ± 0.3	3.9 ± 0.3	3.7 ± 0.3	< 0.001
Cerebral white matter (%)	29.6 ± 1.6	29.7 ± 1.3	27.1 ± 2.1	< 0.001
Cerebellum (%)	9.8 ± 0.7	9.4 ± 0.7	8.9 ± 0.6	< 0.001
Brainstem (%)	3.0 ± 0.2	3.0 ± 0.2	2.9 ± 0.2	0.090
Insula cortex (%)	0.9 ± 0.1	0.89 ± 0.05	0.85 ± 0.06	< 0.001
Hippocampus (%)	0.47 ± 0.03	0.48 ± 0.03	0.47 ± 0.03	0.376
Basal ganglia (%)	0.95 ± 0.09	0.92 ± 0.07	0.97 ± 0.08	0.039
Limbic system (%)	2.38 ± 0.14	2.3 ± 0.1	2.2 ± 0.1	< 0.001
Total ventricle (%)	1.3 ± 0.5	1.5 ± 0.5	2.5 ± 0.8	< 0.001
Lateral ventricle (%)	1.2 ± 0.5	1.3 ± 0.5	2.3 ± 0.8	< 0.001
Third ventricle (%)	0.05 ± 0.02	0.06 ± 0.03	0.11 ± 0.04	< 0.001
Fourth ventricle (%)	0.11 ± 0.02	0.11 ± 0.02	0.12 ± 0.02	0.114
Intracranial CSF space (%)	19.5 ± 2.2	21.5 ± 2.4	27.1 ± 3.0	< 0.001

*p-*value of the differences between the three age groups based on the Kruskal–Wallis rank sum test

## Discussion

Our cross-sectional MRI study using the AI-based automated segmentation method has shown that the brain volume in the healthy subjects began to decline after the 20s and continued to decline over their entire lifespan, with which the CSF volume continued to increase linearly. The mean volume of the intracranial CSF in the 20s was 265 mL, whereas that in the 70s was more than 400 mL. However, the ventricle maintained its volume at approximately 20 mL under the age of 60 years. Although these results in this study are consistent with the results of other volumetric studies [7–16] and our previous MRI studies involving a different population [17–19], they are far from the common knowledge [20–22]: 150 mL in the intracranial CSF and 25 mL in the ventricles in adults. Unless this central dogma is denied, the understanding of the pathophysiology of CSF-related diseases will not progress. Even if the total intracranial CSF volume increases with aging, the ventricles are unlikely to expand in healthy persons under the age of 60 years. After the age of 60 years, the ventricle is more likely to expand for some reasons; for example, the opening foramina of Magendie and Luschka induce increases in the influx of CSF into the ventricle [27–29].

The AI-based automated segmentation has numerous advantages over the conventional voxel-based morphometry or manual 3D segmentation method. The most important advantage is the reproducibility without a spatial normalization technique that fits into a normal brain anatomy on the computer-aided voxel-based morphometry [7–16]. An additional advantage is that the AI-based automated segmentation method takes a short time for analysis (approximately 1 min). Manual 3D segmentation is time-consuming, requires special expertise in anatomical knowledge, and is less reproducible [17–19]. Because the AI-based automated segmentation method using high-resolution brain 3D MRI data is convenient for evaluating brain regional atrophy by referring to the published BrainChart [8], it will be widely clinically applied as a next-generation diagnostic imaging technique, particularly for Alzheimer’s disease.

This study has some limitations. First, the study design was cross-sectional, involving healthy volunteers with wide ranging ages at one point, who were assessed on a 3-tesla MRI machine. Essentially, longitudinal assessments should be ideal to prove the increase in the intracranial CSF volume because of the brain volume loss with aging; however, such studies require a long study period and are difficult to conduct using the same high-resolution MRI machine. Second, in this study, the cognitive function of the participants was not measured, although all volunteers were independent in their daily lives without any problems in walking, writing, memory, and judgment and participated in this study on their own initiative. Third, we had not assessed the reliability of the AI-based automated segmentation volumes obtained using other 1.5-tesla or 3-tesla MRI scanners made by other companies; however, FUJIFILM Corporation verified the aforementioned application. Finally, we assessed the reliability of the segmented volumes in the total ventricles and subarachnoid spaces only, not in the cortical gray and white matter. In our original manual segmentation method, CSF spaces were accurately segmented from 3D T2-weighted images, as previously reported [17–19]. However, segmenting the cortical gray and white matter or the frontal and temporal cortex in 3D is difficult using the manual method. Further work is needed to verify the accuracy of the hippocampal volume obtained using the AI-based automated segmentation method to replace the widespread software for automatic computer-aided voxel-based segmentation systems.

In conclusion, the brain and intracranial CSF could be automatically segmented, and their volumes and volume ratios can be measured within 1 min using a new application worked on the most popular 3D workstation in Japan. This study confirmed the reliability of the volumes segmented using this application. The intracranial CSF volume increased linearly because of the decrease in the brain volume with aging from the 20s to 90s; however, the ventricular volume was maintained until the 60s and then gradually increased. This finding could help elucidate the pathogenesis of chronic hydrocephalus in adults. Furthermore, the volume and volume ratio of the brain could be automatically quantified in 21 subregions. By referring to the BrainChart (http://www.brainchart.io/) [8], the quantitative evaluation of disease-specific brain atrophy based on the segmented brain volume would spread rapidly, and a new era of clinical neuroimaging diagnosis may come in the near future.

## Abbreviations

ADNI: Alzheimer’s Disease Neuroimaging Initiative
AI: Artificial intelligence
CI: Confidential interval
ICC: Intra-class correlation
MPRAGE: Magnetization prepared rapid gradient echo
*P*: Probability
*r*: Pearson’s correlation coefficient
SD: Standard deviation
VSRAD: Voxel-based specific regional analysis system for Alzheimer’s disease

## Declarations

### Guarantor

As this is a multi-center study conducted by the heads of respective institutions, the guarantors of the study are the co-authors. The first and corresponding author, Shigeki Yamada, who is assistant professor (lecture) at the Department of Neurosurgery, Shiga University of Medical Science, will be the primary contact in case of questions.

### Statistics and biometry

SY has significant statistical expertise. He learned biostatistics at the department of the Health and Environmental Sciences, Kyoto University School of Public Health from 2001 to 2004. Since 2003, he was responsible for statistical analysis in more than 30 major papers.

### Study subjects or cohorts overlap

Study subjects in this study did not overlap in our previous publications. All contents in this manuscript have not been published or presented elsewhere in part or entirety and is not under consideration by another journal.

### Methodology

• prospective

• observational/cross sectional study

• performed at one institution
